# Characterization of dFOXO binding sites upstream of the *Insulin Receptor* P2 promoter across the Drosophila phylogeny

**DOI:** 10.1371/journal.pone.0188357

**Published:** 2017-12-04

**Authors:** Dorcas J. Orengo, Montserrat Aguadé, Elvira Juan

**Affiliations:** 1 Departament de Genètica, Microbiologia i Estadística, Facultat de Biologia, Universitat de Barcelona, Barcelona, Spain; 2 Institut de Recerca de la Biodiversitat (IRBio), Universitat de Barcelona, Barcelona, Spain; University of Iceland, ICELAND

## Abstract

The insulin/TOR signal transduction pathway plays a critical role in determining such important traits as body and organ size, metabolic homeostasis and life span. Although this pathway is highly conserved across the animal kingdom, the affected traits can exhibit important differences even between closely related species. Evolutionary studies of regulatory regions require the reliable identification of transcription factor binding sites. Here we have focused on the *Insulin Receptor* (*InR*) expression from its P2 promoter in the Drosophila genus, which in *D*. *melanogaster* is up-regulated by hypophosphorylated Drosophila FOXO (dFOXO). We have finely characterized this transcription factor binding sites *in vitro* along the 1.3 kb region upstream of the *InR* P2 promoter in five Drosophila species. Moreover, we have tested the effect of mutations in the characterized dFOXO sites of *D*. *melanogaster* in transgenic flies. The number of experimentally established binding sites varies across the 1.3 kb region of any particular species, and their distribution also differs among species. In *D*. *melanogaster*, *InR* expression from P2 is differentially affected by dFOXO binding sites at the proximal and distal halves of the species 1.3 kb fragment. The observed uneven distribution of binding sites across this fragment might underlie their differential contribution to regulate *InR* transcription.

## Introduction

**Fig 1:**
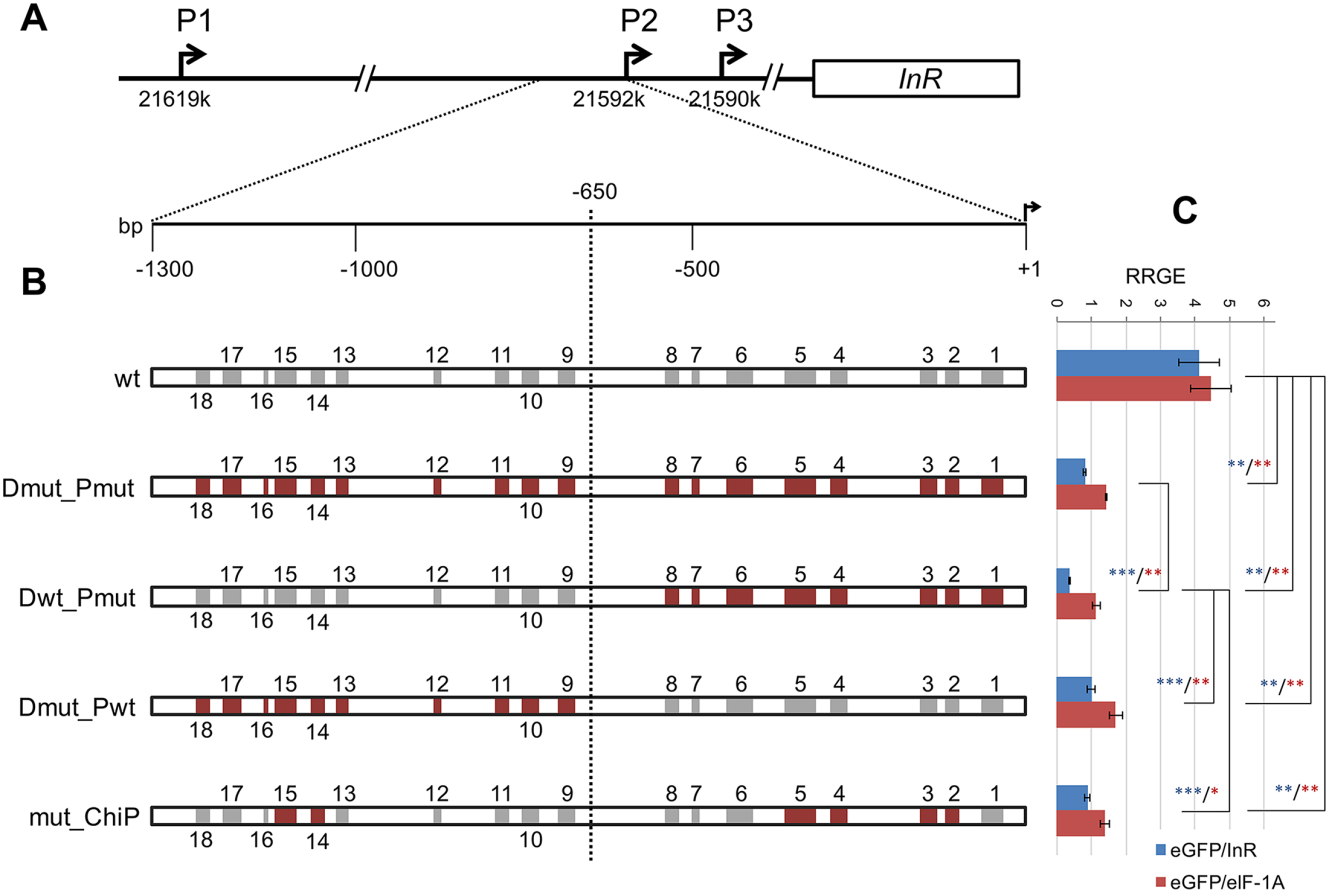
Constructions and analyses of transgenic flies. **(A)** Schematic representation of the *InR* gene and its upstream region in *Drosophila melanogaster* with promoters P1, P2 and P3 coordinates from FlyBase (Release 6). The 1.3 kb fragment upstream of P2 is enlarged to show the location of the dFOXO footprints in this region. **(B)** Schematic representation of the 1.3 kb fragment upstream of the *InR* P2 promoter of wild type and mutated-insert transgenic lines. A discontinuous vertical line indicates the nucleotide position (-650) that divides the fragment into two halves. Grey and brown boxes indicate wild type and mutated footprints, respectively, with their width varying according to the extent of the footprint. **(C)** Expression level of the *eGFP* reporter gene in the different transgenic lines relative to that of the two endogenous genes. RRGE, relative reporter gene expression. * *P*<0.05; ** *P*<0.01; *** *P*<0.001.

The insulin receptor (INR) of the insulin/TOR signal transduction pathway is one of the key sensors of nutrient availability that plays an important role in the control of cellular proliferation, cell size determination, and the response to nutrient availability in metazoans. This pathway is critical for determining body and organ size [1,2]—via regulation of growth and proliferation [3]—as well as metabolic homeostasis and life span [4,5] in *Drosophila*, *Caenorhabditis* and mammals. In *Drosophila melanogaster*, INR constitutes the first step of the insulin/TOR signal transduction pathway. The expression of the *InR* gene is controlled by a set of three distinct promoters (P1, P2, and P3) spread over a rather long region [6] (Fig 1-A). Expression from the P1 promoter that drives the highest basal level of *InR* transcription was initially considered to be stable [6]. Recent work has, however, revealed that the regulation of *InR* expression from P1 is actually more complex as it is influenced by four intronic enhancers, with two of them activating transcription in response to dFOXO and the other two repressing it [7].

In *D*. *melanogaster*, transcription of *InR* from P2 is up-regulated by hypophosphorylated Drosophila FOXO (dFOXO)—a homologue of *Caenorhabditis elegans* DAF-16 and a mammalian orthology group that includes FOXO4 and other duplications (e.g., FOXO1, FOXO3 and FOXO6) [8]. This regulation is conducted through a feedback mechanism triggered by the absence of the insect insulin-like peptides (dILPs) when the nutrients availability is limited [9] and the cellular growth and proliferation are consequently inhibited. dFOXO recognizes, like its homologues, the DAF-16 family-member Binding Element (DBE) and the Insulin Responsive Element (IRE) that had been characterized in the mouse, where their consensus sequences are 5’-TTRTTTKK and 5’-TTRTTTAC, respectively [10–13]. In *D*. *melanogaster*, the bioinformatic analysis of dFOXO targeted regions by ChIP revealed a certain enrichment in FORKHEAD (FKH)-like motifs [14] as well as in the dFOXO binding motif—TKTTYACY—derived from 25 genes [15]. The binding of dFOXO upstream of promoter P2 was partially characterized by band shift analysis, which revealed five 100–150 bp fragments bound by dFOXO spanning a 1.4 kb region. Also occupancy by dFOXO in a 1.5 kb fragment upstream of the P2 promoter has been observed *in vivo* [7,16]. Each of these fragments contains putative FOXO4 recognition elements. This region is also known to respond to dFOXO activation in S2 cells [16].

The insulin/TOR pathway is highly conserved across the animal kingdom. The traits affected by this pathway (*e*. *g*., body size and life span) exhibit, nevertheless, differences even between closely related species of the Drosophila genus [17,18]. Since the number of INR molecules in the cell membrane determines the magnitude of the response to the concentration of nutrients [19], it seems plausible that the number and/or organization of dFOXO binding sites in the *InR* P2 promoter could influence its rate of transcription and consequently, the number of INR molecules that can bind dILPs. This differential binding could underlie the observed differences in the multiple traits affected by the insulin/TOR pathway in Drosophila.

Mutations that modify the activity of transcription factors as well as mutations in the TFBSs can change gene expression and, thus, contribute to evolution. Work in yeast [20], Drosophila [21] and in mice [22] among others suggests that mutations affecting *cis*-regulatory sequences constitute the prevalent source for gene expression divergence between species. However, the comparative analysis of experimentally identified binding sites in some genes of a variety of species has provided substantial evidence for binding site turnover, where the loss of a conserved TFBS would be enabled by the previous gain of a functionally redundant one [23–26]. In those cases where binding site turn over has had no effect on gene expression despite sequence divergence (*e*. *g*., in the Drosophila *eve* stripe 2 enhancer; [27]), stabilizing selection has been proposed to maintain phenotypic constancy and the observed turnover explained by the fixation of neutral compensatory mutations [27]. There is, however, also evidence for positive selection favoring the double mutant [28].

The high turnover of transcription factor binding sites (TFBSs) through evolutionary time [24] and the scarcity of genes with regulatory regions experimentally characterized at the base-pair level, even in most model species and relatives, have so far hampered the development of bioinformatic tools for their identification in a phylogenetic context. Nevertheless, some efforts have been done in this field [29,30]. Only the experimental characterization of the fine structure of the regulatory region of multiple genes in multiple species across a phylogeny might significantly facilitate this endeavor. In Drosophila, most efforts during the last decade have focused: i) on the experimental characterization of TFBSs identified in *D*. *melanogaster* genes and summarized in the DNase I footprint database that does not contain TFBSs for the *InR* gene [31] and ii) on the effect in *D*. *pseudoobscura* and in species of the *melanogaster* group of TFBS turnover in the well characterized *eve* stripe 2 enhancer of *D*.*melanogaster* [27,32]. One major challenge in investigating *cis*-regulatory evolution is the proper alignment of non-coding sequences. This problem has generally been minimized studying closely related sibling species such as *D*. *melanogaster* and *D*. *simulans* [26,28]. Even in this case, the comparative analysis of nucleotide polymorphism and divergence has focused on a set of TFBSs identified by DNase I footprinting since those identified by methods involving genome wide scans, such as ChIP-seq, generally include a large fraction of false positives [28]. DNase I footprinting has also allowed the characterization of the TFBSs present in regulatory regions of distantly related species, which has revealed changes in organization of the ADF-1 binding sites between species of the Sophophora and Drosophila subgenera that had led to temporal changes in *Adh* expression [33].

DNase I footprinting leads to the identification of regions of local DNA protection from DNase I cleavage and it is the method of choice to identify TFBSs at the base-pair resolution level [34] with its automation enabling the scan of long regions. Here, we have identified the dFOXO binding sites at the 1.3 kb fragment upstream of the P2 promoter of the *InR* gene in *D*. *melanogaster* and tested their functionality in transgenic flies. Moreover, the extremely high conservation of the FORKHEAD (FKH) DNA Binding Domain (DBD) of FOXO proteins across the animal kingdom and particularly across the Drosophila genus, has allowed us to use the *D*. *melanogaster* dFOXO protein to experimentally characterize the dFOXO binding sites in the 1.3 kb fragment upstream of the *InR* promoter P2 in four species across the Drosophila phylogeny: three species of the Sophophora subgenus—*D*. *simulans*, *D*. *yakuba* and *D*. *pseudoobscura*—and one species of the Drosophila subgenus—*D*. *virilis*. Our work has revealed that binding sites at the downstream (proximal) half of the *D*. *melanogaster* 1.3 kb fragment have a higher effect on *InR* expression from P2 than those at its upstream (distal) half. It has also revealed i) that the number of binding sites in footprinted sequences varies across the 1.3 kb region of any particular species, and ii) that their distribution differs among species. Our results would therefore suggest that the uneven distribution of dFOXO binding sites across the 1.3 kb fragment detected in *D*. *melanogaster* might underlie the different contribution to *InR* expression from the P2 promoter of sites at the proximal and distal halves of the 1.3 kb fragment in this species, and possibly also across the Drosophila genus.

## Results

### dFOXO footprints in the *Drosophila melanogaster* upstream of the *InR* promoter P2 region

**Fig 2:**
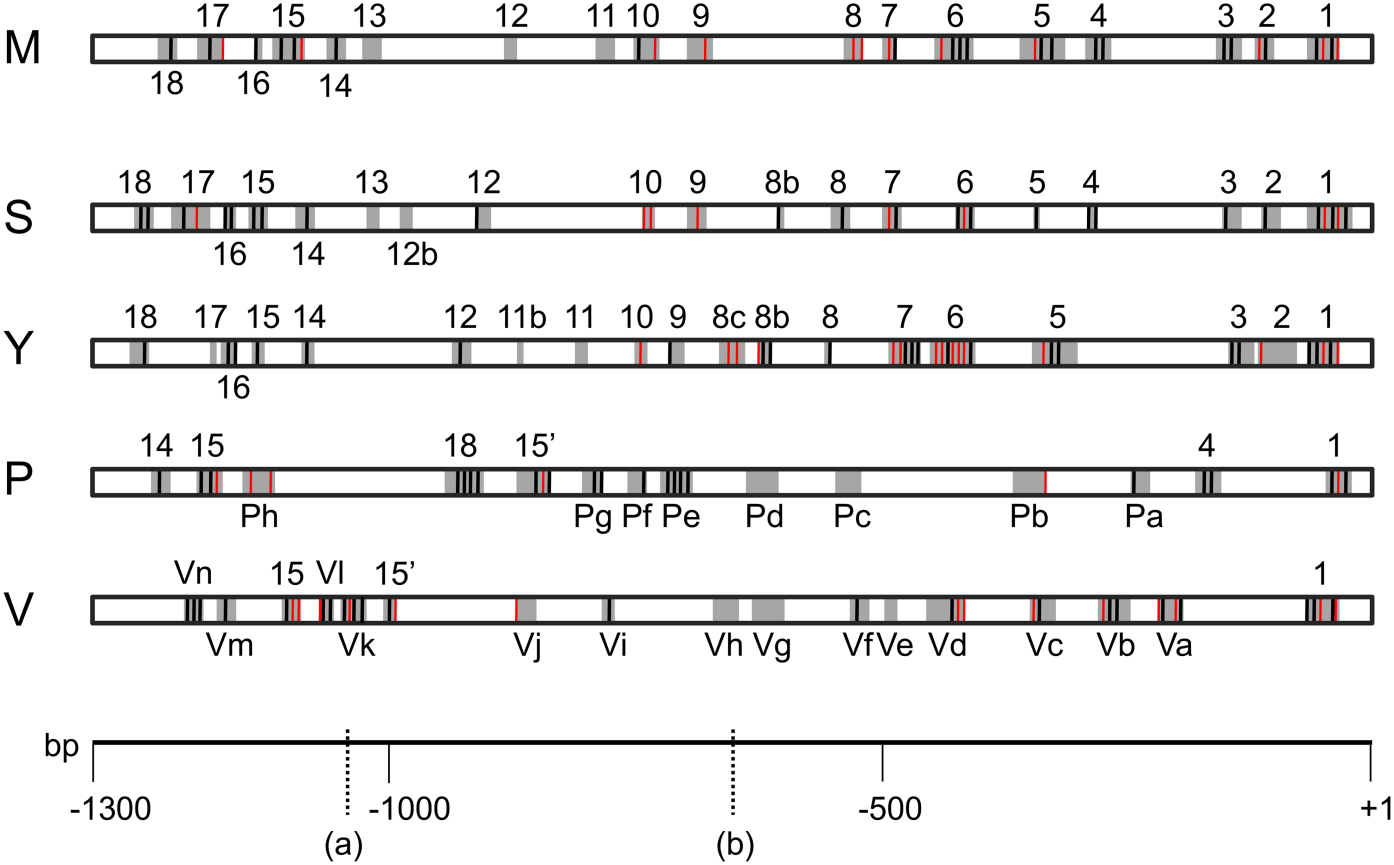
dFOXO footprints upstream of the *InR* P2 promoter across the Drosophila phylogeny. Schematic representation of the 1.3 kb fragment used to identify footprints in each of the five species studied (M, *D*. *melanogaster*; S, *D*. *simulans*; Y, *D*. *yakuba*; P, *D*. *pseudoobscura*; V, *D*. *virilis*). Grey boxes show the location of footprints with their width varying according to the extent of the footprint. Vertical black and red bars indicate the presence of DBE and FKH core sequences, respectively, detected in footprint areas. In species other than *D*. *melanogaster*, footprints that give reliable alignments are indicated with the same number than in *D*. *melanogaster*, with the remaining footprints in *D*. *pseudoobscura* and *D*. *virilis* indicated with the species initial and a correlative small letter. Marks (a) and (b) on the bp ruler correspond to the points used as limits to calculate the local densities of DBE and FKH motifs (-1042 and -650, respectively). Limit (b) was used for that purpose because of its former use in transgenic constructs (see Fig 1). Limit (a) was established at the midpoint between footprints 14 and 13 given that the latter footprint and footprints 12 and 11 are the only ones in *D*. *melanogaster* that do not harbor any core motifs.

In order to understand how dFOXO binding regulates *InR* expression in *D*. *melanogaster*, we finely characterized the *InR* P2 promoter region. DNA footprinting of the 1.3 kb fragment upstream of this promoter was performed using several overlapping fragments (300–500 bp long) covering this region. Each strand of these fragments was FAM labeled and subsequently incubated with either dFOXO or BSA. dFOXO binding sites are protected from DNase I cleavage, which results in clusters of protected residues in each FAM labeled strand that can be identified by a DNA analyzer machine. Automated footprinting of the 1.3 kb region upstream of P2 revealed that dFOXO produces 18 footprints in this region, which include a total of 444 bp. The heterogeneity detected in the number of protected residues within and outside footprints (χ^2^-test, P = 1E-20) would support the threshold used to consider a residue protected (see Materials and methods). These footprints that were named M18 to M1 from the 5’ to 3’ end of the region forward strand (Fig 1-B) differed not only in length—from 9 to 44 bp—but also in the number and distribution of protected sites (S1 Fig). In order to characterize the binding sites of the different footprints, we searched in both the forward and reverse strands for the 5’-TTGTTT and 5’-TTATTT motifs–hereafter named DBE cores–that are shared by the previously identified mouse FOXO binding elements DBE [10,11] and IRE [12,13]. Eight of the here established footprints (M1, M3, M4, M6, M10, M14, M17 and M18) contain the TTGTTT motif, which is repeated either in the same or in different strands of footprints M1 and M6 (Fig 2 and S1 Fig). The TTATTT motif was found in footprints M2, M5 and M15. Moreover, two sequences (TTGTTG and TTTTTT) that deviate by one nucleotide from the consensus DBE and IRE cores were highly protected in two (M5 and M15) and three (M6, M7 and M16) footprints, respectively. We also searched for the 5’-TRTTK core consensus sequence recognized by all FKH proteins [35]. One or more copies of this consensus sequence are present outside the DBE motifs in either the forward or reverse strand, or in both strands (Fig 2 and S2 Fig). It should be noted that footprints M11, M12 and M13 exhibit none of the above reported motifs even though they show clustered protected residues. The DBE and FKH motifs are unevenly distributed across the 1.3 kb region. Indeed, 25% and 66% of these motifs are present, respectively, in the ~0.26 kb and ~0.65 kb most upstream and downstream parts of this region, with the remaining 9% distributed across its ~0.39 kb central part (Fig 2 and S2 Fig).

### Effect of mutations introduced in the dFOXO footprints detected upstream of the *InR* promoter P2 of *Drosophila melanogaster*

The functionality of the detected dFOXO footprints was tested in five transgenic lines that differed by the presence or absence of mutations in protected residues of the footprints reported above (Fig 1-B): wt (wild type), Dmut_Pmut (mutations in all footprints detected, *i*.*e*., in both its distal [D] and proximal [P] halves), Dwt_Pmut (mutations only in the footprints detected in the proximal half of the 1.3 kb fragment), Dmut_Pwt (mutations only in the footprints detected in the distal fragment) and finally mut_ChIP (with mutations in the footprints within the regions previously found to be bound by dFOXO in ChIP experiments [16]). In these lines as well as in the line with only the *eGFP* reporter gene, the segments containing the *miniwhite* and the different versions of the 1.3 kb region controlling the expression of the *eGFP* reporter gene were flanked by *gypsy* insulators [36]. In the different lines, constructs were inserted at the same nucleotide position in section 51C (see Materials and methods). The identical genetic background of all lines and the protection of the reporter gene expression from position effects by flanking *gypsy* insulators [36] set the stage for comparing the different transgenes expression levels. Moreover, examination of the Dmut_Pmut sequence in TRANSFACT revealed that the mutations introduced in the 1.3 kb fragment did not disrupt any binding site for other transcription factors expressed in 22–24 hours embryos (Flybase, http://flybase.org [37]) neither did they generate any new binding site, which would indicate that the effect of the mutations on the different transgenes expression levels would be due to differences in dFOXO binding. The effect of mutations was determined by measuring the expression of the *eGFP* reporter gene relative to that of endogenous genes *InR* and *elF-1A*, in embryos 22–24 hours after egg deposition.

Amplicons for *eGFP*, *InR* and *eIF-1A* have efficiencies ranging from 0.98 to 1.05 (S1 Table) as required to quantify by RT-PCR the reporter (*eGFP*) gene expression relative to a reference gene. In the four lines with mutations in the transgene, the relative expression level of the *eGFP* reporter gene (S2 Table and Fig 1C) is greatly reduced as compared to that of the wild type (wt) transgenic line irrespective of the endogenous gene considered. Indeed, a significant effect of the line (construct) on the reporter gene expression is detected (ANOVA P = 6.1E-9), which can be mainly attributed to differences between the wild type line and the mutated lines (pairwise 2-tailed t-tests, P<0.002 in the four cases). This clearly indicates that at least some, if not all, of the mutations introduced in the dFOXO binding sites of the transgene—in the distal and proximal halves of the 1.3 kb *InR* P2 upstream region—significantly decrease transcription from its P2 promoter. The Dwt_Pmut line exhibits in all cases the most reduced expression of the *eGFP* reporter gene relative to the wt line. Expression is indeed significantly lower in the Dwt_Pmut line than in any other of the three mutated transgenic lines (1-tailed t-tests, P<0.001 for the 3 comparisons with *InR* as reference gene, and P equal to 0.007, 0.005 and 0.034 for those with *eIF-1A*). The significantly more reduced expression detected in the Dwt_Pmut than in the Dmut_Pwt line points to a lesser role of the dFOXO binding sites located in the distal half of the 1.3 kb *InR* P2 upstream region on levels of expression from this promoter. Moreover, the significantly more reduced expression detected in the Dwt_Pmut line than in either the Dmut_Pmut or mut_ChIP lines would suggest that in the latter lines the effect of mutations in the distal half of the 1.3 kb region might compensate for the effect of mutations in its proximal half.

### dFOXO binding sites across the Drosophila phylogeny

The multiple alignment of the 110-aminoacid dFOXO DNA-binding domain sequences [38] of the five species here studied–*D*. *melanogaster*, *D*. *simulans*, *D*. *yakuba*, *D*. *pseudoobscura* and *D*. *virilis*–revealed their identity except for one amino-acid difference in *D*. *virilis*. The high conservation detected allowed us to use the *D*. *melanogaster* protein for the automated footprinting of the 1.3 kb region upstream of the *InR* P2 promoter in each of the other four Drosophila species that exhibit increasing times of divergence to *D*. *melanogaster*. The number of footprints produced by dFOXO in these four species is 19, 19, 14 and 17, respectively (Fig 2 and S1 Fig). These footprints include a total of 387, 417, 405 and 405 bp, respectively. Sequences of the 1.3 kb region upstream of the P2 promoter could only be reliably aligned along their complete length in the three species of the *melanogaster* group (S3 Fig). In *D*. *pseudoobcura* and *D*. *virilis*, only small regions that encompass some of the detected footprints could be reliably aligned with the remaining sequences through BLAST search (S3 Fig). The MultiZ alignments on the genome UCSC browser (http://genome.ucsc.edu) of the 1.3 kb fragment confirmed these results except for the distal part of *D*. *pseudoobscura* sequence that is inverted in this species. Footprints and binding motifs in reliably aligned regions can be considered homologous but not those in other regions. Homologous footprints are numbered according to the *D*. *melanogaster* footprint number (Fig 2). In the *melanogaster* group species, footprint position is conserved in 15 out of 18 cases, and an additional aligned footprint is shared by *D*. *simulans* and *D*. *yakuba*. In *D*. *pseudoobscura*, footprint sequences P1 and P4 align with M1 and M4 in the same position of the multiple alignment whereas the fragment spanning footprints P14, P15 and P18 is inverted relative to that of the three *melanogaster* group sequences, and footprint P15 is present twice (labeled P15 and P15’). In *D*. *virilis*, only two footprints (V1 and V15) can be confidently aligned with all other sequences, and V15 is also present twice (labeled V15 and V15’).

In order to characterize the binding sites of the different footprints, we searched for the presence of DBE core motifs as well as of FKH consensus sequences (S2 Fig). In the three species of the *melanogaster* group, nine footprints (1, 3, 5, 6, 7, 14, 15, 16 and 18) have DBE motifs in the three species but only in five footprints (1, 3, 14, 15 and 16) are these motifs aligned in all three. Relative to *D*. *melanogaster*, four and eight of the footprinted regions exhibit an additional DBE motif in *D*. *simulans* (S8, S12, S16 and S18) and *D*. *yakuba* (Y6, Y7, Y8, Y8b, Y9, Y12, Y16 and Y18) respectively, whereas three in *D*. *simulans* (S5, S6 and S10) and six in *D*. *yakuba* (Y2, Y6, Y7, Y10, Y15 and Y17) exhibit one less. In *D*. *pseudoobscura*, DBE motifs are present in the six alignable footprinted regions (P1, P4, P15’, P18, P15 and P14) and in four of the eight non-alignable footprints (Pa, Pe, Pf and Pg). Also in *D*. *virilis*, they are present in the three alignable footprinted regions (V1, V15 and V15’) and in ten of the fourteen non-alignable footprints (Va, Vb, Vc, Vf, Vi, Vj, Vk, Vl, Vm and Vn). Furthermore, and as shown in Fig 2, some footprints contain additional FKH motifs.

The number of DBE core motifs detected in the 1.3 kb region of the five species with characterized dFOXO footprints ranges from 20 to 22, and that of the additional FKH motifs from 6 to 15 (Fig 2, S2 and S4 Figs). These motifs are unevenly distributed along the 1.3 kb region. In the three species of the *melanogaster* group, the central part of this region exhibits the lowest density of motifs (Table 1). The relative density of motifs in its two flanking segments varies among the three species: rather similar in *D*. *melanogaster* and *D*. *simulans* but not in *D*. *yakuba*. In *D*. *virilis*, the density of motifs is also lowest in the central part of the 1.3 kb region, but its most upstream part exhibits a much higher motif density than any of the *melanogaster* group species do. The distribution of DBE and FKH motifs across the 1.3 kb region of *D*. *pseudoobscura* is the most discordant as it exhibits the highest density of motifs in its central part.

**Table 1:** Densities of DBE and FKH motifs in three fragments of the 1.3 kb region.

	Fragment 1* 258 bp	Fragment 2 392 bp	Fragment 3 650 bp
*D*. *melanogaster*	31.0	7.7	32.3
*D*. *simulans*	34.9	10.2	26.2
*D*. *yakuba*	19.4	10.2	43.1
*D*. *pseudoobscura*	23.3	35.7	10.8
*D*. *virilis*	46.5	15.3	27.7

Values correspond to the number of DBE+FKH motifs per kb.

*See Fig 2 for fragment limits

From 105 DBE sites experimentally detected in the five species here analyzed, a new Drosophila DBE consensus sequence can be derived: 5’-TTDTTKNB (S4 Fig). Interestingly, our results indicate that dFOXO can recognize this motif with a thymine base at position 3 and also that thymine is the most frequent base in the two nucleotides at the 3’ end of the motif. This motif differs at some positions from that based in a collection of dFOXO ChIPed genes not including *InR* that were analyzed in adult females—5’-TKTTYMCY—[15].

## Discussion

The identification of the TFBSs contained in *cis*-regulatory elements constitutes the first step to elucidate the genetic and molecular mechanisms responsible for *cis*-regulatory divergence among species. We focused on the regulation of the *InR* gene transcription from its P2 promoter, despite that its transcript yield is much lower than from its P1 promoter, because a significant direct activation of P2 by dFOXO has been confirmed both *in vitro* and *in vivo* [7,16] whereas dFOXO also indirectly activates or represses additional enhancers located within introns of *InR* gene [7]. Previous band-shift studies had shown that in *D*. *melanogaster* dFOXO binds to four fragments upstream of this promoter [16], but these studies provided no sequence information on the actual binding sites. The automated DNase I footprinting here performed circumvents this limitation as it allows the identification of TFBSs at the base-pair resolution level *in vitro*. Our work revealed multiple dFOXO binding sites in each of the fragments previously found to be bound by dFOXO *in vitro*. Additionally, our footprinting results allowed us to uncover, relative to the band-shift work, eight new and strong footprints: five distal footprints (M14 to M18) and three proximal footprints (M1 to M3) flanking the TATA box. It should be, however, added that footprints M14 and M15 are in the region that had been previously reported to be bound by dFOXO *in vivo* [16]. Most of the characterized footprints contain sequences TTRTTT (S2 Fig) as in the mouse DBE consensus sequence [10,11] but footprints containing TTTTTK were also found to be strongly protected from DNase I digestion. The observed nucleotide differences between the motif sequences present in the footprints and in the mouse consensus core sequence could be plausibly explained by the amino acid substitutions that have occurred since the divergence of the mouse and Drosophila lineages outside the totally conserved canonical FOXO base-contacting residues in the recognition helix. Thus, conformational rearrangements [39] of the dFOXO DBD would have led to the modified consensus recognition sequence 5’-TTDTTKNN for Drosophila dFOXO. According to Lynch and Hagner [40], such variation in TFBSs is expected to be a natural consequence of the degrees of freedom associated with binding interfaces, the diminishing advantages of increased affinity and the limits to the power of natural selection.

In *D*. *melanogaster*, FKH core sequence TRTTK is present not only in some footprints with DBE but also in some footprints lacking this element. The distribution of DBE and FKH core motifs is not homogeneous across the 1.3 kb region. Indeed, its most proximal (~0.65 kb) and distal (~0.26 kb) parts exhibit higher densities of DBE motifs (Fig 2, S2 Fig, and Table 1) than the central part (~0.39 kb). When the motifs in the 0.65 kb proximal half were mutated, the expression of the *eGFP* reporter gene in transgenic *D*. *melanogaster* flies was even lower than that of transgenic flies carrying mutated motifs along the complete 1.3 kb region. However, the expression of the reporter gene relative to endogenous genes was in all cases lower in transgenic flies with mutated motifs only in the proximal half of the 1.3 kb region than in those with mutated motifs only in its distal half. This result would indicate that even though the normal expression of the *InR* gene from promoter P2 requires motifs with the consensus sequence in the distal half, these motifs have a lesser effect on transcription from this promoter than motifs present in the proximal half, which might be related to their relative numbers in both segments. Moreover, transgenic flies with mutated motifs only in the proximal half exhibit the lowest level of reporter expression relative to the *InR* and *eIF-1A* endogenous genes (Fig 1-C). This observation suggests that at least some mutations introduced in the distal half might compensate the effect on gene expression of mutations introduced in the proximal half. The minor differences observed in the relative expression of the reporter gene between transgenic lines with mutated motifs in all footprints (Dmut_Pmut) and those with a subset of mutated motifs in both the distal and proximal halves (mut_ChIP) suggests i) that the compensatory mutations would be either in the M14 or M15 footprints, and ii) that mutations in footprints M1, M6 and M7 might play a major role in the extreme reduction detected in transgenic flies with mutated motifs only in the proximal half (Dwt_Pmut). The presence of an nc-RNA gene embedded in the 1.3 kb region used in transgenic constructs might raise the question of its putative effect through transcriptional interference in our *in vivo* results. Although the lack of information on how this nc-RNA gene expression is regulated, and more importantly on where its regulatory elements are located, precludes evaluating this possibility, its putative interference might only have a minor effect on our results. Indeed, any effect on the endogenous *InR* gene transcription would be similar in the five transgenic lines.

The characterization of DBE motifs by DNase I footprinting upstream of the *InR* P2 promoter in *D*. *melanogaster* and the subsequent ascertainment that mutations in these motifs reduce transcription from this promoter led us to characterize the corresponding dFOXO binding sites in a 1.3 kb fragment upstream of the P2 promoter in four additional species across the Drosophila genus in order to get new insights in binding site evolution. The 1.3 kb fragment of the five species studied can be considered homologous despite that only parts of the *D*. *pseudoobscura* and *D*. *virilis* sequences could be reliably aligned with those of the three species of the *melanogaster* group because i) its proximal part that encompasses the TATA box can be reliably aligned throughout the five species, ii) the distal part of the *D*. *pseudoobscura* sequence can be reliably aligned when reverse-complemented given that it is inverted in this species, and iii) the homology revealed by the MultiZ alignment (http://genome.ucsc.edu) across the five species at the beginning of the second exon of the nc-RNA gene that is present ~300-bp from the 1.3 kb distal end.

The number of DBE and FKH core motifs in footprinted sequences varies among the five species studied from 27 in *D*. *pseudoobscura* to 36 in *D*. *virilis*. These motifs distribution is rather uneven both within any given species and among species. The core motifs detected in footprinted sequences are most similarly distributed in *D*. *melanogaster* and *D*. *simulans*. In these species as well as in *D*. *yakuba* and *D*. *virilis*, it is the central part of the 1.3 kb region that exhibits the lowest density of motifs (Fig 2, S2 Fig and Table 1). In *D*. *pseudoobscura*, the DBE and FKH core motifs distribution differs from that observed in the other four species. The comparison of dFOXO binding sites in the region upstream of the P2 promoter across the five species studied has revealed that only two of them are conserved across the Drosophila genus, whereas 15 are only conserved in the more closely related species of the *melanogaster* group. Although TFBS conservation across distantly related species is most easily explained by the action of purifying selection, both stabilizing selection and positive selection have been proposed to underlie TFBS turnover [27,28]. It should be, however, noted that one limitation of footprinting analysis by automated DNAse I is that not all bound sites by a transcription factor *in vitro* are necessarily also bound *in vivo*. Our work sets the ground for additional experiments with transgenic flies carrying mutations in the dFOXO binding sites detected *in vitro* in the four additional species, which would reveal if and how their distribution affects the expression and feedback regulation [16] of the *InR* gene from promoter P2 in those species.

In summary, our experimental characterization of dFOXO binding sites upstream of the *InR* P2 promoter in five *Drosophila* species with different divergence times has revealed that their number varies among species. Most importantly, it has shown that they exhibit an uneven distribution along the 1.3 kb fragment studied, distribution that is only similar in the three more closely related species, with *D*. *pseudoobscura* exhibiting an even more discordant pattern relative to these species than *D*. *virilis* does. Also, our analysis of expression in transgenic flies with mutations at subsets of the dFOXO binding sites upstream of the gene P2 promoter in *D*. *melanogaster* has revealed that *InR* expression is more affected in this species by binding sites at the proximal than at the distal half of the 1.3 kb fragment studied. The uneven distribution of DBE motifs might account, at least partly, for this differential effect.

## Material and methods

### Drosophila strains and sequences

The *D*. *melanogaster*, *D*. *simulans*, *D*. *yakuba*, *D*. *pseudoobscura* and *D*. *virilis* strains (accession nos. 14021–0231.36, 14021–251.216, 14021–261.01, 14011–0121.94 and 15010–1051.87, respectively) used in the initial Drosophila genome projects [41–43] were obtained from the UCSD Drosophila species stock center. The orthologous sequences of the *InR* promoter 2 upstream region as well as the orthologous sequences of the dFOXO DNA-binding domain were obtained from FlyBase (http://flybase.org) [37].

### DNase I footprinting

#### Cloning and sequencing

For each of the five species, an ~3 kb fragment upstream of the *InR* P2 promoter was amplified from purified DNA (QIAGEN, Hilden, Germany) using the AmpliTaq Gold polymerase (AB Applied Biosystems, Thermo Fisher Scientific, Waltham, MA, USA), and primers designed to directionally clone the fragment in the pET101/D-TOPO plasmid vector (Invitrogen, Thermo Fisher Scientific, Waltham, MA, USA). From each species clone, overlapping 300 to 500 bp long subclones were obtained through the amplification of the corresponding fragment with AmpliTaq Gold polymerase and suitable primers (S3 Table) and its subsequent cloning in the same vector.

All clones and subclones inserts were sequenced with the ABI PRISM version 3.2 cycle-sequencing kit (AB Applied Biosystems) according to manufacturer’s conditions. Sequencing products were separated on an ABI PRISM 3730 sequencer (Applied Biosystems). All sequences were obtained on both strands and assembled using the DNASTAR package [44]. Sequences have been deposited in the EMBL/GenBank Data Libraries under accession numbers LT838814-LT838818.

#### Automated footprinting

The four steps required for automated footprinting [45] are described below with the modifications introduced in the present study.

For FAM labeling (step 1), forward (5’-AGGGTTAGGGATAGGCTTACCT) and reverse (5’-AGCGGATAACAATTCCCCTCTA) primers were designed to anneal at the pet101/D-TOPO vector regions flanking the subcloned insert. 5’-6 FAM labeled and unlabeled primers were synthesized by SIGMA (St. Louis, MO, USA). In order to label each fragment at either end, fragments were amplified alternating which primer was 5’-6-FAM labeled. PCR reactions were performed at the same conditions for all fragments (50 μl total reaction volume, dNTP 2.5 mM each, MgCl_2_ 25mM, PCR buffer II, Amplitaq Gold 0.4 μl, forward and reverse primers 10 μM each, DNA from the corresponding subclone 5 ng; 95°C 5 min., 35 cycles 95°C 15 sec., 58°C 15 sec., 65°C 30 sec., and 65°C 7 min.). The amplified fragments were purified with QIAquick PCR columns (QIAGEN) and its concentration quantified with Qubit (Invitrogen).

Binding reactions (step 2) were performed in a 20 μl total volume using a modified version of the Brent (2008) buffer (5% glycerol, 0.2 mM EDTA, 50 mM KCl, 2 mM MgCl_2_, 20 mM Tris HCl pH = 8, 0.2 mM DTT) and 20nM DNA. Four reactions were performed: two with BSA at either a 600 nM or 1200 nM concentration, and two with dFOXO purified by GenScript (Piscataway, NJ, USA) at either of those concentrations. Reactions were incubated at 20°C for 30 min.

DNase I reactions (step 3) were performed by adding 20 μl of DNase I dilution buffer (10 mMTris-HCl, 10 mM CaCl_2_, 10 mM MgCl_2_, 10% Glicerol) containing 0.03 Katz units of DNase I (Amersham, GE Healthcare Life Sciences) to each binding reaction and posterior incubation at 20°C for 1 min. Reactions were stopped with 40 μl of 0.5 M EDTA and placed on ice. DNA was extracted with phenol: chloroform, chloroform and purified with a QIAquick PCR column (QIAGEN). Digested DNA concentration was quantified with Qubit and diluted to 0.6 ng/μl.

Automated footprinting (step 4) was performed adding 3.75 μl autoclaved water, 5.85 μl HiDiFormamide and 0.15 μl 600 LIZ (Applied Biosystems) to 1.25 μl of digested DNA. Five replicates of each DNA sample were run in an ABIPRISM 3730 DNA analyzer (injection time 30 sec. and voltage 3.0 kV) with the Genemapper50_POP7 and DFACE software. Results were analyzed with the Peak Scanner (Applied Biosystems) version 1.0 software package (S1-S25 Datasets and S5 Fig). As all the 5’FAM_forward labeled and 5’FAM_reverse labeled fragments have, respectively, the first 69 and the last 48 vector residues in common, the peaks corresponding to the first and last residues of the *InR* fragment are easily identified in the electropherograms. Upon exporting the sizes and heights provided for all peaks in each sample, those of each sample were aligned with the corresponding fragment sequence using a script designed for that purpose. This script aligns the sequence of the fragment with the position (size) of each peak across the five replicates of each sample both for standards and experimental DNA, and it then calculates the mean height for each peak. The mean height of all standard peaks in a particular sample was used to normalize the values of mean height of each experimental peak in that sample. The BSA/dFOXO ratio was calculated for each peak. In order to define a footprint, it should be first noted that DNase I does not recognize sequence *per se* and its cutting rates vary along a given DNA sequence depending on global variation in helix groove width and radial asymmetry, and local variation in phosphate accessibility [46]. It should be also noted that the observed footprinted sequence in one strand can overlap or be slightly offset from that in the other strand. We therefore considered protection at both strands and defined a footprint according to the following criteria: i) taking into account that a BSA/dFOXO ratio value equal to one means no protection, only residues with a BSA/dFOXO ratio ≥ 2 in the experiments performed with either 600 nM or 1200 nM protein concentrations were considered to be protected, ii) protected residues had to be clustered, iii) when the footprinted sequence in one strand overlapped that in the complementary strand, the footprint extent was defined in the strand with the longest footprinted sequence; and iv) when the footprinted sequences in both strands were slightly offset, the limits of the footprint were defined by the 5’ protected residues of each strand.

### Construction of transgenic lines and expression analysis

The pGreen Rabbit reporter vector that contains insulator (*gypsy*) elements—effective in preventing position effects—flanking the *mini-white* gene, and the reporter *eGFP* gene [36] was used for cloning and PhiC31 integrase-mediated transgenesis. Three types of recombinant vectors were obtained according to their insert: i) with no insert, ii) with the *D*. *melanogaster* wild type 1.3 kb fragment upstream of the *InR* P2 transcription start site (TSS), and iii) with different mutated versions of the 1.3 kb fragment. Sequences with changes relative to the wild type were synthesized by Gene Script (NJ, USA).

The different transgenic lines were generated by Genetic Services (MA, USA) by injection into the attP 51D platform line. For each transgenic line, insert location was verified through PCR amplification—using primers flanking the integration site (at section 51D) and the corresponding insert—, and subsequently sequencing the amplification product. Insert identity was similarly verified through its PCR amplification and subsequent sequencing (Accession numbers LT838819 –LT838822).

Real Time RT-PCR was used for gene expression quantification taking into account MIQUE guidelines [47]. Total RNA was purified from 22–24 hours embryos developed at 22°C using the RNeasy Plus Mini kit (QIAGEN). This stage was chosen because it shows the highest expression of the endogenous *InR* and *dFoxo* genes (modENCODE Development RNA-Seq in FlyBase) [37]. An additional step of on-column DNase I, RNase-free digestion was introduced into the procedure to get RNA free of DNA. RNA integrity was established with Bioanalizer (Agilent Technologies, CA, USA) and RNA concentration was determined with Qubit (Invitrogen, Thermo Fisher Scientific). The optimal primers and MGB probes (S4 Table) were designed with the Primer Express Program (Applied Biosystems, Foster City, CA, USA).

Three technical replicates of transgenic line RNA samples were analyzed in a 7900HT Sequence Detection System (Applied Biosystems). The RNA samples were first diluted to 200 ng / 4.43 μl. Subsequently, either four or three 1:4 dilutions were obtained depending on the construct. In each experiment, the standard curve for each gene was obtained from the pGR line, using four total RNA dilutions (equivalent to 200, 50, 12.5 and 3.125 ng of total RNA per sample) (S5 Table). Amplification efficiencies for each gene were estimated using three dilutions (equivalent to 200, 50 and 12.5 ng of total RNA per sample, S1 Table). For any particular gene, transcript amount in transgenic lines was calculated using 200 ng total RNA and the standard curve generated for the same gene with total RNA from line pGR (S5 Table). Real Time RT-PCR was performed with the TaqMan RNA-to CT kit (Applied Biosystems, Thermo Fisher Scientific, Waltham, MA, USA). Reactions of 14 μl total volume contained 900 nM of each primer, 250 nM MGB probe, 4.43 μl of the appropriate RNA dilution and amounts of Taqman RT-PCR mix and Taqman Enzyme mix as indicated by the supplier. In order to normalize the level of expression of the *eGFP* reporter gene in each transgenic line, the expression of two endogenous genes (*InR* and *elF-1A*) was also measured. The expression of the *InR* gene from promoter P2 was determined because the 1.3 kb region upstream of this promoter should bind the same transcription factors (other than dFOXO) than the *InR* insert in the tested constructions, which would therefore allow to correct for any minor differences in developmental stage that might occur among embryo samples. Endogenous gene *elF-1A*, was used because of its previously reported high expression stability value [48].

### Software used

The MUSCLE software was initially performed to obtain the multiple alignment of the 1.3 kb fragment in the five species. This alignment was refined manually with the help of the outputs obtained from the Align Sequences Nucleotide BLAST utility (NCBI webpage) that was used to search for similarities between the *D*. *melanogaster* footprinted sequences and those of the other species. Indeed, results from BLAST, allowed us to notice that *D*. *pseudoobscura* has a microinversion in this sequence.

The Peak Scanner (Applied Biosystems) version 1.0 software package was used to display the electropherogram images (S5 Fig) and to export the sizes and heights provided for all peaks in each sample. Visual inspection of the superimposed images of electropherograms for the BSA and FOXO experimental conditions allowed a preliminary identification of the footprinted sequences that was then confirmed by the precise comparison of their peak relative heights. For this purpose, a couple of homemade R scripts were used (available upon request).

One- and two-tailed t tests as well as ANOVA were performed using the R package [49].

## Supporting information

S1 Data Numerical outputs from Peak Scanner. (ZIP)

S1 Fig Sequences of the dFOXO footprints detected in the 1.3 kb region upstream of the *InR* P2 promoter in five Drosophila species. (PDF)

S2 Fig DBE and FKH core motifs identified in the dFOXO footprints of Drosophila. (PDF)

S3 Fig Multiple sequence alignment of the 1.3 kb fragment upstream of the *InR* P2 promoter in five Drosophila species. (PDF)

S4 Fig DBE motifs in five species of the Drosophila genus. (PDF)

S5 Fig Fragment of an electropherogram including a dFOXO footprint. (PDF)

S1 Table Amplicon efficiencies in RT-PCR experiments. (PDF)

S2 Table eGFP expression relative to the endogenous genes. (PDF)

S3 Table Oligonucleotides used to amplify the different fragments upstream of the *InR* P2 promoter that were cloned into the pET101/D-TOPO vector. (PDF)

S4 Table Oligonucleotides used for RT-PCR experiments. (PDF)

S5 Table Results of RT-PCR experiments. (PDF)
