# Supplementary material for: Characterization of dFOXO binding sites upstream of the Insulin Receptor P2 promoter across the Drosophila phylogeny
Source: PLoS One. 2017 Dec 4;12(12):e0188357. doi: 10.1371/journal.pone.0188357 (PMC5714339; doi:10.1371/journal.pone.0188357)
Supplement: S1 Fig — (PDF) [file pone.0188357.s002.pdf]

S1 Figure. Sequence of the dFOXO footprints detected in the 1.3 kb region upstream of the *InR* P2 promoter in five Drosophila species.

A) *D. melanogaster* dFOXO footprints

```
M18    -1231 CCCTATTTGTTTATTCAATTCCG -1209
             GGGATAAACAAATAAGTTAAGGC

M17    -1196 GTGTTTTTGTAGTTGTTTGTGCTATTACG -1168
             CACAAAAACATCAACAAACACGATAATGC

M16    -1137 TTTTTTTTT -1129
             AAAAAAAAA

M15    -1112 TTTGTTGTCTAATTTATTTCTATTTCTA -1085
             AAACAACAGATTAAATAAAGATAAAGAT

M14    -1061 TTGAAAAACAAATTACC -1045
             AACTTTTTGTTTAATGG

M13    -1024 GTTATGCATTAATTGTAA -1007
             CAATACGTAATTAACATT

M12    -885 TGCATTAATTCTTC -872
            ACGTAATTAAGAAG

M11    -790 CTGCAAAATTGATTTTATTA -771
            GACGTTTTAACTAAAATAAT

M10    -749 CAAACAATTGGATAGTCATAAATATTA -723
            GTTTGTTAACCTATCAGTATTTATAAT

M9     -697 CGGGTTTTAAGCATAAACATTTACATGG -670
            GCCCAAAATTCGTATTTGTAAATGTACC

M8 *   -535 GATTAAAAATACACCTATTT -516
            CTAATTTTTATGTGAATAAA

M7     -498 ATATGCACTGTTTTTT -483
            TATACGTGACAAAAAA

M6     -446 ACATATCTATTTTGTAAAACAATTTTTTGTCTTTGTTTTCC -406
            TGTATAGATAAAACATTTTGTTAAAAAACAGAAACAAAAGG

M5     -355 GAATCTAGGTGTTTTTGTTGCTAATCTGGTTATTTACTGTACTC -312
            CTTAGATCCACAAAAACAACGATTAGACCAATAAATGACATGAG

M4     -289 CGTTCTTGTTGTTTTTGCTCGGAACT -264
            GCAAGAACAACAAAAACGAGCCTTGA

M3     -153 TTTCATTGTTGTTTGTATTATTATTACT -126
            AAAGTAACAACAAACATAATAATAATGA

M2     -118 CTGTTGTTATTTGGCAGAAGCA -97
            GACAACAATAAACCGTCTTCGT

M1     -66 ATGAAGGAAAAACAATATTGTGTCTTGTTTATTGC -32
           TACTTCCTTTTTGTTATAACACAGAACAAATAACG
```

B) *D. simulans* dFOXO footprints

S18
-1261 T C C T T A T T T G T T T A T T C A A T T C C G -1238
A G G A A T A A A C A A A T A A G T T A A G G C

S17
-1222 T T T T T G T A G T T G T T T G T G C T A T T A C G T A T T G T A A A A T T G A A A -1181
A A A A A C A T C A A C A A A C A C G A T A A T G C A T A A C A T T T T A A C T T T

S16
-1169 G T T G T T T T T T G T G T G C -1154
C A A C A A A A A A C A C A C G

S15
-1144 A T T T G T T G T C T A A T T T A T T T C T -1123
T A A A C A A C A G A T T A A A T A A A G A

S14
-1099 C C C C A G A T T G A A A A A C A A A T T A C C -1076
G G G G T C T A A C T T T T T G T T T A A T G G

S13
-1049 C A T T A A T T G T A A T A G A -1034
G T A A T T A A C A T T A T C T

S12b
-991 C A C A C A A T T A A G A G -978
G T G T G T T A A T T C T C

S12
-910 A A A T A A A T C A A T T C -897
T T T A T T T A G T T A A G

S10
-742 A A A T A T T A A A C A T T -729
T T T A T A A T T T G T A A

S9
-700 T T T C A A G C A T A A A C A T T T A C A T G G -677
A A A G T T C G T A T T T G T A A A T G T A C C

S8b
-602 T T G T T T T C -595
A A C A A A A G

S8
-549 A A A T T A A A G T T A T T T C T T G A -530
T T T A A T T T C A A T A A A G A A C T

S7
-496 A A T T T G T A T T T T T T T T T A A T T -476
T T A A A C A T A A A A A A A A A T T A A

S6
-425 A T T T T T T G T A T T T G T T T T C C T A T -403
T A A A A A A C A T A A A C A A A A G G A T A

S5
-343 T T T T G T T G -336
A A A A C A A C

S4
-284 T T G T T G T T T T -275
A A C A A C A A A A

S3
-147 T G T T G T T T G T A T T A T T A T T A -128
A C A A C A A A C A T A A T A A T A A T

S2
-112 T T A T T T G G C A G A A G C A G G -95
A A T A A A C C G T C T T C G T C C

S1
TATA
-66 A A G A A G G A A A A A C A A T A T T G T G T C T T G T T T A T T G C T T T A T T T G C G G C G T -18
T T C T T C C T T T T G T T A T A A C A C A G A A C A A A T A A C G A A A T A A A C G C C G C A

C) *D. yakuba* dFOXO footprints

Footprint	Start	Sequence (top / bottom strand)	End
Y18	-1265	GATTAGTTTATTAGTTTATTTA<br>CTAATCAAATAATCAAATAAAT	-1244
Y17	-1180	AATTGAAAC<br>TTAACTTTG	-1172
Y16	-1162	TGTTGTTTTTTG<br>ACAACAAAAAAC	-1151
Y15	-1136	AATTGTTGTCTAATG<br>TTAACAACAGATTAC	-1122
Y14	-1090	TGAAAAACAAATTAC<br>ACTTTTTGTTTAATG	-1076
Y12	-933	aaagaaaataaatgaatt<br>TTTCTTTTATTTACTTAA	-916
Y11b	-871	gaaatc<br>CTTTAG	-866
Y11	-806	atttaatttat<br>TAAATTAAATA	-796
Y10	-749	attaaacatttgac<br>TAATTTGTAAACTG	-736
Y9 *	-712	aaacaacacactt<br>TTTGTTGTGTGAA	-700
Y8c	-663	gaaatgttgtgtattattatcaaatag<br>CTTTACAACACATAATAATAGTTTATC	-637
Y8b	-625	ATCAATATTGTTTTTT<br>TAGTTATAACAAAAAA	-610
Y8 *	-559	TAATTATTT<br>ATTAATAAA	-551
Y7	-494	TTGTATAAGAAAAATATTTGTTGTTGATTTGTTTTGG<br>AACATATTCTTTTTATAAACAACAACTAAACAAAACC	-458
Y6	-451	AAAACATACATATTTATTTTGTAAAATATTTTTCTGTATTTGTTTTC<br>TTTTGTATGTATAAATAAAACATTTTATAAAAAGACATAAACAAAAG	-405
Y5	-347	CGAATCTAGGTGTTTTTGTTGCTAATCTGGTTATTTACTGTACTTTAGT<br>GCTTAGATCCACAAAAACAACGATTAGACCAATAAATGACATGAAATCA	-299
Y3	-148	TCATTGTTGTTTGTATTATTATTACTCGT<br>AGTAACAACAAACATAATAATAATGAGCA	-120
Y2	-117	TGCTGTTGTTATTAGGCAGAAGCAAGGAATAAAGGAGAGGA<br>ACGACAACAATAATCCGTCTTCGTTCCTTATTTCCTCTCCT	-77
Y1	-62	AGAAAAAACAATATTGTGTCTTGTTTATTGCT<br>TCTTTTTTGTTATAACACAGAACAAATAACGA	-31

D) ***D. pseudoobcura*** **dFOXO footprints**

```
P14    -1241  G G T A A T T T G T T T T T C A A T C T G  -1221
              C C A T T A A A C A A A A A G T T A G A C

P15    -1194  T A G A A A T A A A T T A G A C A A C A A C A C A A  -1169
              A T C T T T A T T T A A T C T G T T G T T G T G T T

Ph *   -1156  A T T T T T A C A A T A C G T A A T A A C A A A A G C A G C A A C A  -1123
              T A A A A A T G T T A T G C A T T A T T G T T T T C G T C G T T G T

P18    -945   G A G C A G G A G A A T T G A A T A A A C A A A C G A C A A C A A C A A A A A A A T C  -904
              C T C G T C C T C T T A A C T T A T T T G T T T G C T G T T G T T G T T T T T T T A G

P15' * -871   A G A A C A A G A G C C A C A A C A A T A A T A A A A A T A G A A A T A A  -835
              T C T T G T T C T C G G T G T T G T T A T T A T T T T T A T C T T T A T T

Pg     -804   G A C A G A A C G C A T C A A C A A C A A A  -783
              C T G T C T T G C G T A G T T G T T G T T T

Pf     -755   A C G G C A A A A A A A A A A A T T  -738
              T G C C G T T T T T T T T T T T A A

Pe     -722   A C G A A A G A A A A A A A C G C A A C A A C A A A A G C A A A C A A A  -687
              T G C T T T C T T T T T T T G C G T T G T T G T T T T C G T T T G T T T

Pd     -634   A T T C A T A C T A T T A A A T T A A A T T A A G A A A T T C C T  -602
              T A A G T A T G A T A A T T T A A T T T A A T T C T T T A A G G A

Pc     -546   A T G G G A T A C C T T T A T A A G C A A A C C T T C  -520
              T A C C C T A T G G A A A T A T T C G T T T G G A A G

Pb *   -373   G A T C G G A A A G A T C G G T A A G A A T A C A A A T T G T A T A T T T  -337
              C T A G C C T T T C T A G C C A T T C T T A T G T T T A A C A T A T A A A

Pa     -247   A T T T G T T G T A C C T T T T C T T T C A C  -226
              T A A A C A A C A T G G A A A A G A A C C G

P4     -179   T G C T C T G G T T G T T T T T G T T G T C C C T G G A A C  -150
              A C G A G A C C A A C A A A A A C A A C A G G G A C C T T G
                                          TATA
P1     -46    C G T C T T G T T T A T T G C T T T A T T T G C G G C  -20
              G C A G A A C A A A T A A C G A A A T A A A C G C C G
```

E) *D. virilis* dFOXO footprints

Footprint	Start	Sequence (top strand / bottom strand)	End
Vn *	-1203	ATTTTGTTTTTTTCTTTTTT<br>TAAAACAAAAAAAGAAAAAA	-1184
Vm	-1174	TGTTATTGTTTAAGTTTTCT<br>ACAATAACAAATTCAAAAGA	-1155
V15 *	-1109	CGTTTTATTTCTATTTTTATTG<br>GCAAAATAAAGATAAAAAATAAC	-1088
Vl	-1068	ATATTGTTGTTGTAAC<br>TATAACAACAACATTG	-1053
Vk	-1047	CTTGTTGTTATTGTTGATTTTTTTCA<br>GAACAACAATAACAACTAAAAAAAGT	-1022
V15' *	-1002	ATTTATTTCTATTT<br>TAAATAAAGATAAA	-989
Vj	-871	AAACATAACAGAGCAAATTAAGAG<br>TTTGTATTGTCTCGTTTAATTCTC	-848
Vi	-779	caaaaacaaaa<br>GTTTTTGTTTT	-769
Vh	-667	atacacacacacacacaaacgagt<br>TATGTGTGTGTGTGTGTTTGCTCA	-644
Vg	-626	AAAACTTGAGCATTATGAAGCACGTTGGCGTT<br>TTTTGAACTCGTAATACTTCGTGCAACCGCAA	-595
Vf	-528	GAAAATCACAACAATAATCAG<br>CTTTTAGTGTTGTTATTAGTC	-508
Ve	-497	TGAACAACTAATTTTAA<br>ACTTGTTGATTAAAATT	-481
Vd *	-455	TATGTGTCTTTCTAGTAAATAATTAATTCAAAAATTAAAATATTG<br>ATACACAGAAAGATCATTTATTAATTAAGTTTTTAATTTTATAAC	-411
Vc	-344	CTCTGTTGTTTTCTTCCACATGTTC<br>GAGACAACAAAAGAAGGTGTACAAG	-320
Vb	-284	TCCAAACGTGTTGTTGTTGTTGTGCATGTTCATTCA<br>AGGTTTGCACAACAACAACAACACGTACAAGTAAGT	-249
Va	-219	TATTTTTTTTTCGTTTTCTGTTTATTTA<br>ATAAAAAAAAAGCAAAAGACAAATAAAT	-192
V1	-62	AAAAAAACAATATTGTGTCTTGTTTATTGCT<br>TTTTTTTGTTATAACACAGAACAAATAACGA	-32

Footprints are named as indicated in Figure 2. The sequences of fragments protected by dFOXO are indicated for both strands. Protected residues are highlighted in dark orange if protected in experiments performed at both 600 nM and 1200 nM concentrations of dFOXO, and in light orange if they are at only one of these concentrations. In each footprint, the DBE core consensus is indicated in large-font red letters whereas additional FKH cores are indicated in small red letters. Black horizontal lines above or below residues highlight overlapping DBE core motifs. An asterisk indicates that one or more non-protected nucleotides have been added to the footprinted sequence to complete a DBE core motif. Lower case letters with grey shadowing of one footprint strand indicates that protected residues were not assessed in that strand.
