# Supplementary material for: Characterization of dFOXO binding sites upstream of the Insulin Receptor P2 promoter across the Drosophila phylogeny
Source: PLoS One. 2017 Dec 4;12(12):e0188357. doi: 10.1371/journal.pone.0188357 (PMC5714339; doi:10.1371/journal.pone.0188357)

S2 Figure. DBE and FKH core motifs identified in the dFOXO footprints of Drosophila. The footprints of *D. melanogaster* are numbered as in Fig. 1 and Supplementary Fig. S1. The footprints that are reliably aligned (Supplementary Fig. S3) are indicated with the same number in the five species. In *D. pseudoobscura* and *D. virilis* footprints that did not align are indicated with the species initial and a correlative small letter. DBE core motifs and additional FKH core motifs are indicated as black and red circles, respectively. Motifs that overlap in a given footprint are shadowed in blue.

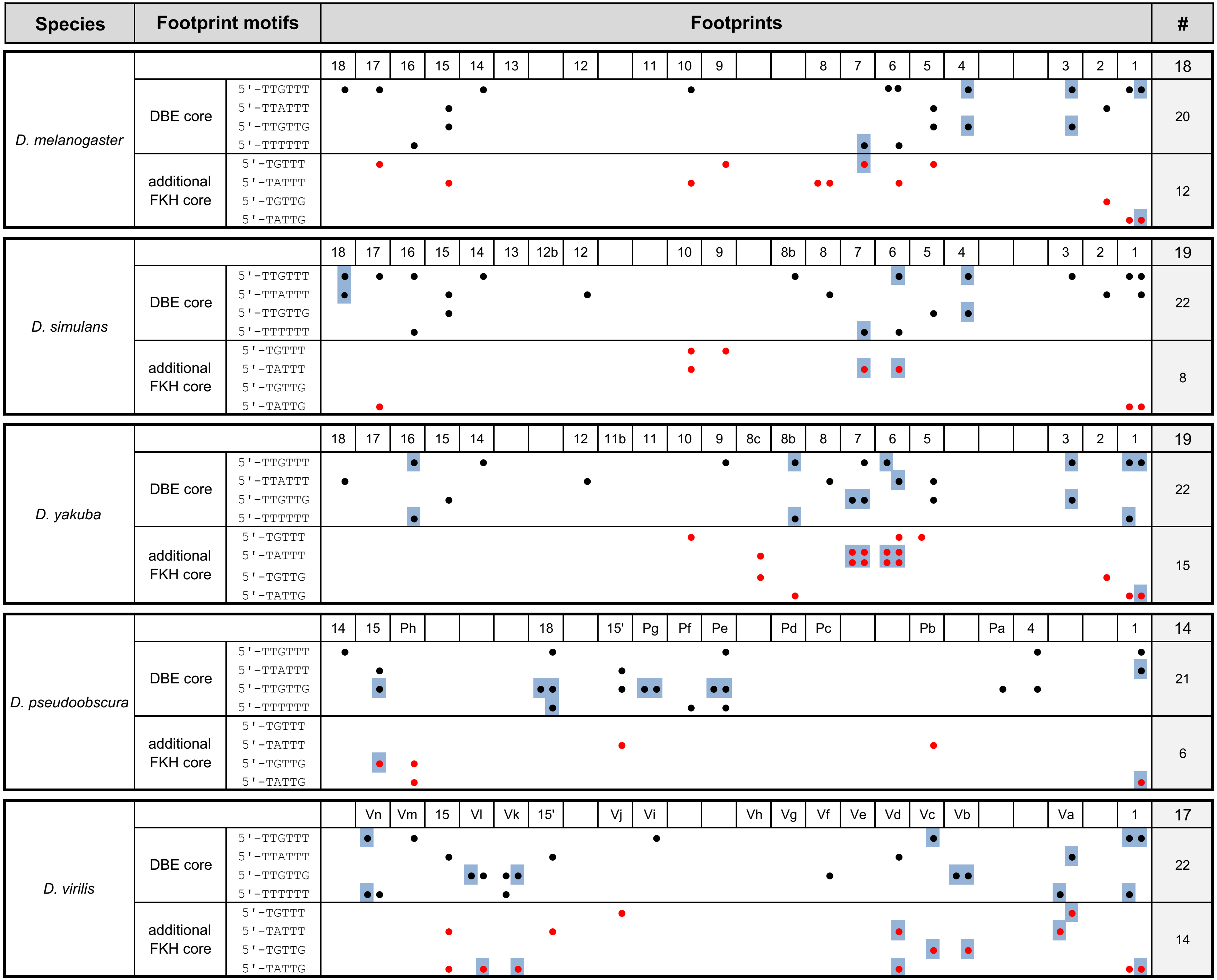

Supplement: S2 Fig — (PDF) [file pone.0188357.s003.pdf]
