# Supplementary material for: Characterization of dFOXO binding sites upstream of the Insulin Receptor P2 promoter across the Drosophila phylogeny
Source: PLoS One. 2017 Dec 4;12(12):e0188357. doi: 10.1371/journal.pone.0188357 (PMC5714339; doi:10.1371/journal.pone.0188357)
Supplement: S3 Fig — (PDF) [file pone.0188357.s004.pdf]

S3 Figure. Multiple sequence alignment of the 1.3 kb fragment upstream of the *InR* P2 promoter in five Drosophila species

```
                                                                                                   #18
ref         TTATTTTTTGCCTATGGTTCGTTTTGTATTTCTATTTCTTATT-AC-GACTTTT-GCGCTTTGTTGCTTTTCCCTATTTGTTTATT-------------CAATTCCGCCACTGATACCGG
mel [-1300] ...........................................-..-.......-................CCCTATTTGTTTATT-------------CAATTCCG............G  [-1196]
sim [-1329] ...................t.....................c.-..-.......-...............TCCtTATTTGTTTATT-------------CAATTCCG.............  [-1226]
yak [-1336] .....gca.ct..c.a...t........-.g......t...c.-..-....a..-....c.c.....c...t.tgATTaGTTTATTagtttatttagttt.......t............  [-1220]
pse [ -886]                           // .c..a..at..Ag.Cg.TGAtTTTTTG---TTgtTgtCg--------TTTGTTTATT-------------CAATTCt-CCtgctc....tc //  [ -952]

                          #17                                                 #16                                  #15
ref         TGTTTTT---GTAGTTGTTTGTGCTATTACGTATTGTAAAATTGAAAGCGTCTTCTGTGTTTTTTTTTTTGTGTGCCCGCTGCCTATTTGTTGTCTAATTTATTTCTATTTCTACGCCTA
mel         TGTTTTT---GTAGTTGTTTGTGCTATTACG.............................TTTTTTTTTT................TTTGTTGTCTAATTTATTTCTATTTCTA......  [-1079]
sim         ..TTTTT---GTAGTTGTTTGTGCTATTACGTATTGTAAAATTGAAA...........G--TTgTTTTTTGTGTGC.........ATTTGTTGTCTAATTTATTTCT.............  [-1111]
yak         a....g.gta.......c............a.....c..AATTGAAAc.......g.TG--TTgTTTTTTG.aaa...c..a...AaTTGTTGTCTAATg....................  [-1102]
pse [-1116] ..c.a..---..t.c..c..t..t.................a..t.--.......-----------a.TTGTG--------------TTGTTGTCTAATTTATTTCTA..........gc  [-1206]
pse [ -820]                                                                                  // .ta.t...t..gtt.TTATTTCTATTTtTAttatTg  [ -854]
vir [-1016]                                                                               // ...c...g........ATTTATTTCTATTT.....t..c  [ -978]
vir [-1109]                                                                                             // CgtTTTATTTCTATTTtTAttg..g  [-1085]

                                    #14                                 #13
ref         TA-TGGTATGTCCCCAGATTGAAAAACAAATTACCATAAGTGTCGTTTTGTAAGTGTTATGCATTAATTGTAATAGAAATATTGCTGTAACGCTCAAAAGCAAAAGC---------ATCCA
mel         ..-...............TTGAAAAACAAATTACC....................GTTATGCATTAATTGTAA..................................---------.....  [ -968]
sim         ..-.a......CCCCAGATTGAAAAACAAATTACC......................a...CATTAATTGTAATAGA................c............taaaaagtaa...CA  [ -991]
yak         ..---------..t...c.TGAAAAACAAATTAC...........------------------.............................atca....g..t...---------g...g  [-1016]
pse [-1207] cgt....tg....tCAGATTGAAAAACAAATTACC....a.....⏏........t...a...t..........t..c........t..c.g.--------a....a.---------.....//  [-1331]
pse [ -855] TtgTGGctctTgttCt //  [ -871]
vir [ -977] gc-.acg.a.ct.g.........................a....ag.......t...a.................ct............ //  [ -890]

                                                                                                   #12
ref         CACAATTAGGAGATTTCTCCGCAGTAGTTGTGCTCTTCA--AACCAG----CAAAGTAATCAGTGCTCATC--TCCCGAGAGAAAGGAGATGCATTAATTCTTCTTTTTCTTCTACGAGG
mel         .......................................--......----....................--.................TGCATTAATTCTTC................  [ -856]
sim         CACAATTAaGAG........a..................--....c.----....................--..............AaATaaATcAATTC....c....gc.......a  [ -879]
yak         t.a...g.at.....a...........c..a....agt.at..g.t.agca...........a.......tt..........AAAGaAaATaaATgAATT.....c.c..g.....cg.a  [ -896]

                                                                                                 #11
ref         AAAT-GCGAGAGTGCGGCTTTGTAGAAATCTTGCGAGAATCTT----------TT------TTTAGTTTAAATCTTAGAAAACTGCAAAATTGATTTTATTATAGAGTTCCTAGAAAGCT
mel         ....-......................................----------..------.....................CTGCAAAATTGATTTTATTA..................  [ -752]
sim         .tc.-......................................gaacagtatc..------...tag.....................................................  [ -765]
yak         .tc.caaa.....t.....cgaa.GAAATC.c....t......gcgcacagta..tgtata....a.a.....................ATTtAaTTTAT....................  [ -776]

                          #10                                                        #9
ref         TGGCAAACAATTGGATAGTCATAAATATTAAACACTTGAC-----ACAGCTTCTGAGAATCGGGTTTTAAGCATAAACATTTACATGGCTTCTGTGAAGTATAGTTTCTTGTAAATGTAA
mel         ...CAAACAATTGGATAGTCATAAATATTA..........-----...............CGGGTTTTAAGCATAAACATTTACATGG................................  [ -637]
sim         g.............c.......AAATATTAAACAtT....ttgac.a........g........TTTcAAGCATAAACATTTACATGG......g.......................g.  [ -645]
yak         ..a.......................ATTAAACAtTTGAC-----....t.........c..a.c..cAAaCA-AcACAcTT...c.t......g.......................gA  [ -662]
```

```
                                                                                                                        
ref          AATGTTGTG---TTTTATCAAATAATGAATGGATGACGAATTAAAAATGGTTTTCTCTGT----------CGGTATAAAACTTCTGTGTTTAAATGTGGAAGAATATCGTTTACCGATTA
mel          .........---................................................----------.............................................GATTA    [ -531]
sim          .........---...............--..t......c........TtGTTTTC.....----------...........................a..-----------......a..    [ -551]
yak          AATGTTGTGtatTaTTATCAAATAg----..t......c.aTcAAtATtGTTTTtT....cacatgctgc.a....g.........a.........aa.---------------------    [ -566]

                 #8                                                             #7
ref          AAAATACACCTATTTTCTTGAGT-----------------------GGTTT--------TAAAATATGCACTGTTTTTTTTAA---------TTATTGTTAATTTGTTTTCTTACCCTTT
mel          AAAATACACCTATT.........-----------------------.....--------....ATATGCACTGTTTTTT....---------............................    [ -451]
sim          AAAATtaAagTtaTTTCTTGAa.tccccaaattatttaaaaagttg.t...--------.t.AATtTGtAt--TTTTTTTTAA---------TT.................a....t...    [ -450]
yak          g.....tAatTATTT...a...ctctccaaaattgactgtaaaacgta..cgtcattgg.c....t..t..----TTgTaTAAGAAAAATATTTgTTGTTgATTTGTTTTgg...ttaaa    [ -450]

                                             #6                                                                     #5
ref          AA-GCACATATCTATTTTGTAAAACAATTTTTTGTCTTTGTTTTCCTTTGGTCTTGCT----AGTTTAGCTGCGTCTGTCTGTCTGCCT---CGTCTCGTCGCGAATCTAGGTGTTTTTG
mel          ..-..ACATATCTATTTTGTAAAACAATTTTTTGTCTTTGTTTTCC............----...........................---...........GAATCTAGGTGTTTTTG    [ -339]
sim          ..-a....c................-ATTTTTTGTaTTGTTTTCCTaT.ac.......----...........................---.......................TTTTG    [ -339]
yak          AAcatACATATtTATTTTGTAAAAtAtTTTTcTGTaTTGTTTTC.....c......aaaa...a......................t..gcc...a......CGAATCTAGGTGTTTTTG    [ -330]

                 #5(continued)                                #4
ref        TTGCTAATCTGGTTATTTACTGTACTCTAGTAGTTGCTCTTGTGTCTCTCGTTCTTGTTGTTTTTGCTCGGAACTG-AACTGGAGCTTT--GAGAATCGAGATTTCAGCTTCGAGAGACCGAG
mel        TTGCTAATCTGGTTATTTACTGTACTC......................CGTTCTTGTTGTTTTTGCTCGGAACT.-...........--................................    [ -219]
sim        TTG.......................t......................gc...TTGTTGTTTT............-............--..........................g..a..    [ -219]
yak        TTGCTAATCTGGTTATTTACTGTACTtTAGT....t.........a...g.gc..........c............-......g.....--..............t........------..    [ -216]
pse   [ -186]                            //     a.c...---tGcTCTgGTTGTTTTTGtT-GtccCTGGAAC.........TT.....-.-..gg.  //                [ -123]

                                                                                             #3                               #2
ref          ACTGAGACTGGATGGAAATGGAAACTACTGGCACTGTTTGGGCAGCTATTTCGAAACCGCTCTCGTTTCATTGTTGTTTGTATTATTATTACTCGTTTTGCTGTTGTTATTTGGCAGAAG
mel          .................................................................TTTCATTGTTGTTTGTATTATTATTACT.......CTGTTGTTATTTGGCAGAAG    [  -99]
sim          .......................................................................TGTTGTTTGTATTATTATTA...............TTATTTGGCAGAAG    [  -99]
yak          .......g....................................ag.....................TCATTGTTGTTTGTATTATTATTACTCGT..TGCTGTTGTTATTaGGCAGAAG    [  -96]

                                                                          #1                 TATAbox
ref          CAAGGAATAAAGGAGAGGAGAAGAAGTAGCAGATGAAGGAAAAACAATATTGTGTCTTGTTTATTGC[TTTATTT]GCGGCGTTTT-GCTCTTGATTAGGTT
mel          CA..............................ATGAAGGAAAAACAATATTGTGTCTTGTTTATTGC[.......]..........-...............    [ +1]
sim          CAgG.......................g....AaGAAGGAAAAACAATATTGTGTCTTGTTTATTGC[TTTATTT]GCGGCGT...-...............    [ +1]
yak          CAAGGAATAAAGGAGAGGA...---..t.a...a.gAGaAAAAACAATATTGTGTCTTGTTTATTGC[T......]..........-...............    [ +1]
pse   [-80] //..ga.gggtg...g..a.g..g.gg.gg.ag.g-------------------.cGTCTTGTTTATTGC[TTTATTT]GCGGC.....-...............    [ +1]
vir   [-73]               //.....a..-------------a..aaAAAAACAATATTGTGTCTTGTTTATTGC[T......]..........g...............    [ +1]
```

ref, reference sequence; mel, *D. melanogaster*; sim, *D. simulans*; yak, *D. yakuba*; pse, *D. pseudoobscura*; vir, *D. virilis*.
In order to facilitate footprint identification and comparison across species, the *D. melanogaster* sequence was used both as reference (ref) and as part of the alignment. Numbers above the reference sequence refer to the *D. melanogaster* footprints. Sequence coordinates relative to the transcription start site from the P2 promoter are indicated within square brackets, and highlighted in red in those cases where sequences were reverse-complemented prior to their alignment. Experimentally established footprints are highlighted in yellow and their sequence explicitly given in each compared species. Red letters refer to DBE cores identified either in the forward or reverse (underlined) strand of the footprinted sequence. Lowercase letters show those nucleotides different from the reference sequence. Dots represent non-protected conserved nucleotides. Dashes refer to InDels in the alignment. Slashes indicate breaks in sequence contiguity. A ⏏ symbol refers to a 22-nucleotides insertion.
