# Supplementary material for: Characterization of dFOXO binding sites upstream of the Insulin Receptor P2 promoter across the Drosophila phylogeny
Source: PLoS One. 2017 Dec 4;12(12):e0188357. doi: 10.1371/journal.pone.0188357 (PMC5714339; doi:10.1371/journal.pone.0188357)
Supplement: S4 Fig — (PDF) [file pone.0188357.s005.pdf]

S4 Figure. DBE motifs in five species of the Drosophila genus.

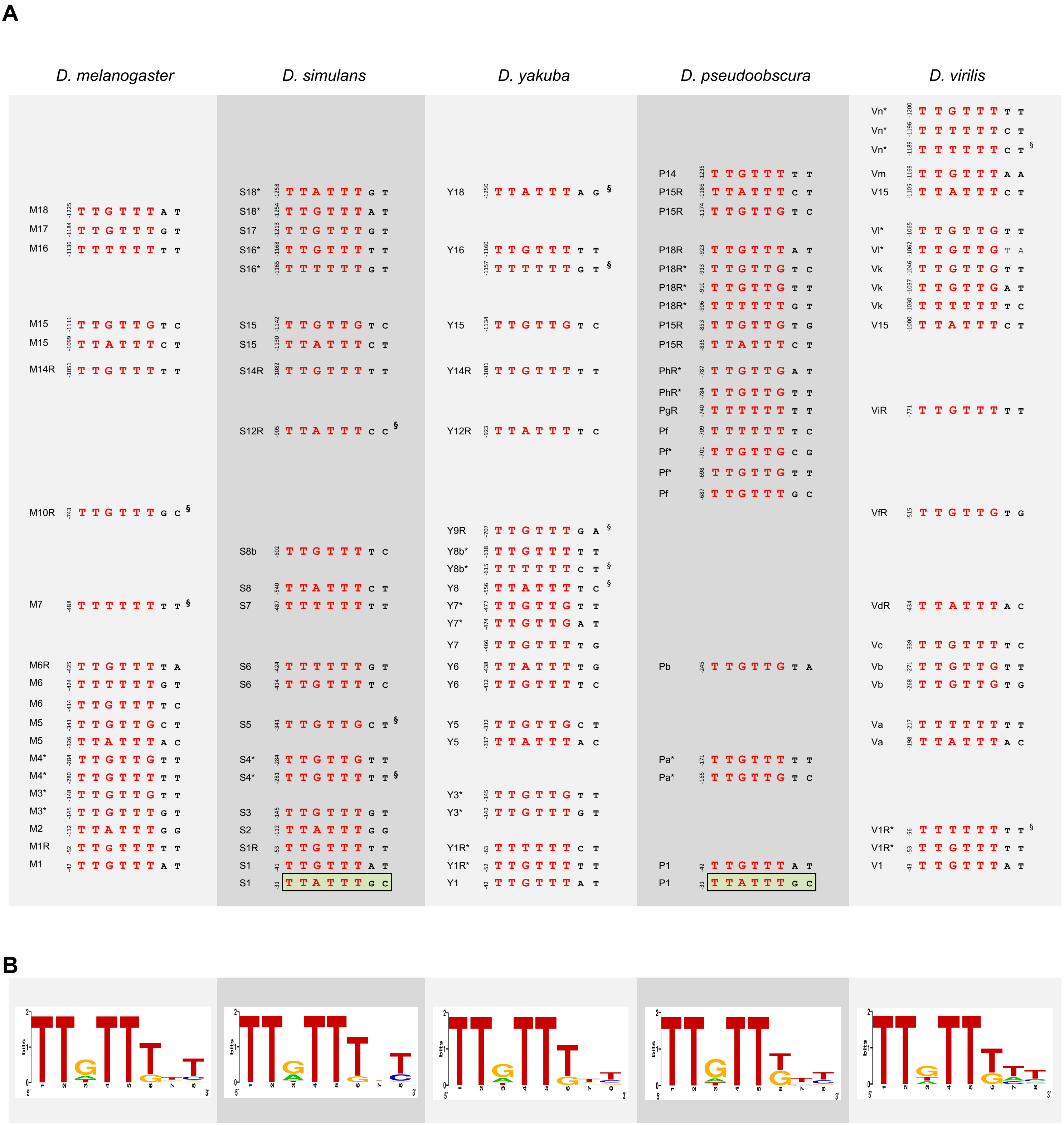

A. Sequences of DBEs present in dFOXO footprints. Red letters refer to the core motif, and black letters to the remaining two nucleotides. R refers to motifs present in the reverse strand.

* overlapping motifs

§ The complete DBE sequence (eight nucleotides) was considered to obtain the element consensus sequence in each species as well as in the Drosophila genus, eventhough some of the last nucleotides are not protected.

DBE that overlaps theTATA box

B. DBE consensus sequence in each species.
