# Supplementary material for: Characterization of dFOXO binding sites upstream of the Insulin Receptor P2 promoter across the Drosophila phylogeny
Source: PLoS One. 2017 Dec 4;12(12):e0188357. doi: 10.1371/journal.pone.0188357 (PMC5714339; doi:10.1371/journal.pone.0188357)
Supplement: S5 Fig — (PDF) [file pone.0188357.s006.pdf]

S5 Figure. Fragment of an electropherogram including a dFOXO footprint.

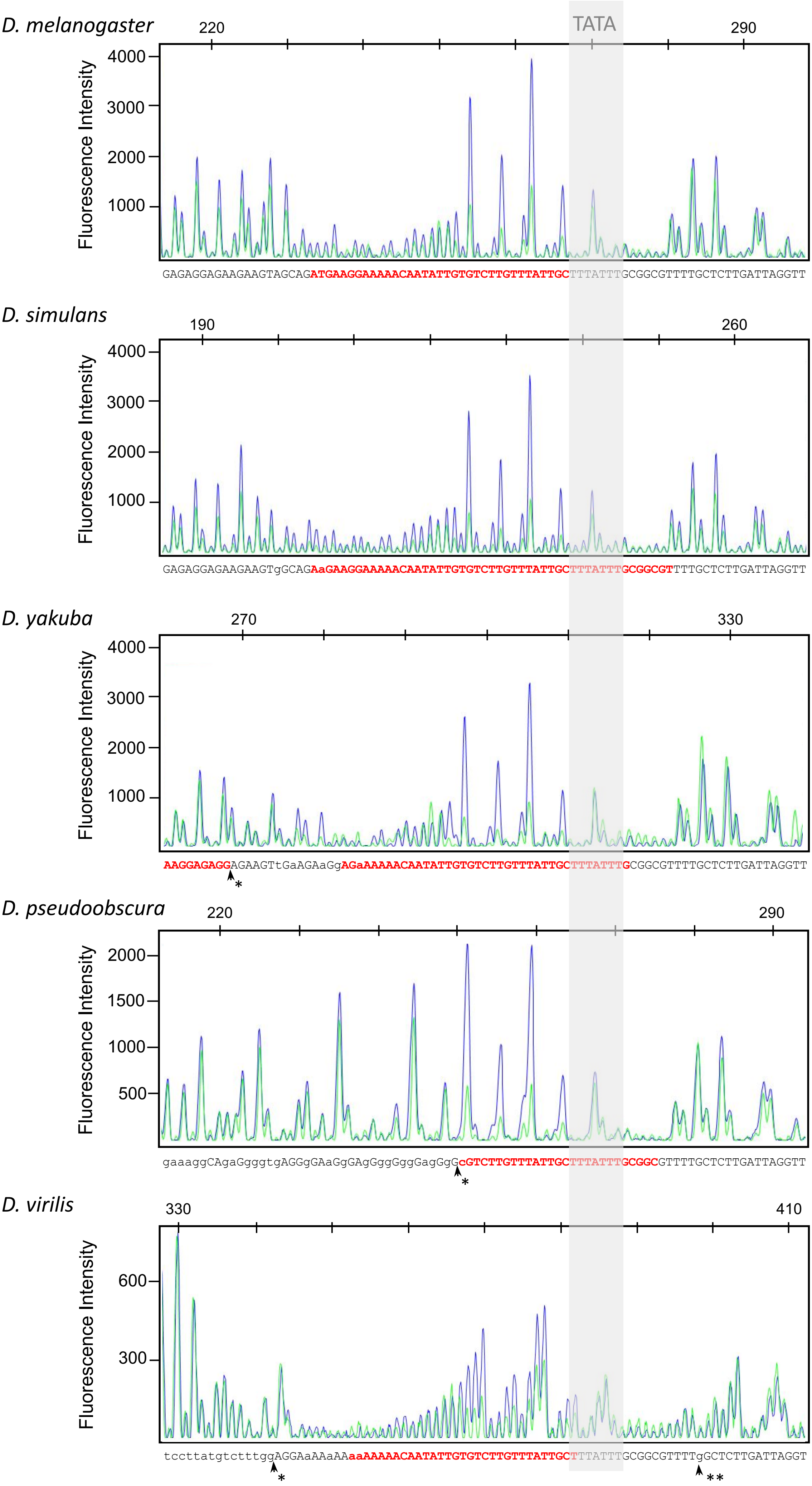

Blue and green superimposed lines correspond to one replicate of the BSA and dFOXO experiments, respectively. Numbers above electropherograms show the nucleotide position in the cloned fragment. The fluorescence intensity decreases with the length of fragments (right end of the electropherogram) although the resolution is good. Below each electropherogram, the corresponding nucleotide sequence is shown. A gray shaded region highlights the TATA box. Lowercase letters indicate nucleotide changes relative to the *D. melanogaster* sequence. Red letters show the footprint identified in this fragment where, for multiple nucleotide residues, green peaks are significantly lower than blue peaks, and thus revealing that dFOXO protected DNA from DNase I digestion (footprint delimitation was based on 5 replicates of each the forward and reverse strands of the fragment). An arrowhead indicates the location of either a deletion (*) or an insertion (**) relative to the *D. melanogaster* sequence.
