# Supplementary material for: Characterization of dFOXO binding sites upstream of the Insulin Receptor P2 promoter across the Drosophila phylogeny
Source: PLoS One. 2017 Dec 4;12(12):e0188357. doi: 10.1371/journal.pone.0188357 (PMC5714339; doi:10.1371/journal.pone.0188357)
Supplement: S1 Table — (PDF) [file pone.0188357.s007.pdf]

S1 Table. Amplicon efficiencies in RT-PCR experiments

	Efficiency = $\mathbf{10^{(-1/slope)} -1}$		
Line	*eGFP*	*InR*	*eIF-1A*
wt	1.052	1.055	0.957
Dmut_Pmut	1.054	1.034	1.006
Dwt_Pmut	1.020	1.046	0.971
Dmut_Pwt	0.963	1.038	0.973
mut_ChIP	0.964	1.062	0.972
