# Supplementary material for: Characterization of dFOXO binding sites upstream of the Insulin Receptor P2 promoter across the Drosophila phylogeny
Source: PLoS One. 2017 Dec 4;12(12):e0188357. doi: 10.1371/journal.pone.0188357 (PMC5714339; doi:10.1371/journal.pone.0188357)
Supplement: S2 Table — (PDF) [file pone.0188357.s008.pdf]

S2 Table. *eGFP* expression relative to endogenous genes

Line	*eGFP/InR*	*eGFP/eIF-1A*
wt	4.096	4.439
Dmut_Pmut	0.815	1.433
Dwt_Pmut	0.361	1.129
Dmut_Pwt	0.990	1.705
mut_ChIP	0.888	1.384
