# Supplementary material for: Characterization of dFOXO binding sites upstream of the Insulin Receptor P2 promoter across the Drosophila phylogeny
Source: PLoS One. 2017 Dec 4;12(12):e0188357. doi: 10.1371/journal.pone.0188357 (PMC5714339; doi:10.1371/journal.pone.0188357)
Supplement: S3 Table — (PDF) [file pone.0188357.s009.pdf]

S3 Table. Oligonucleotides used to amplify the different fragments upstream of the *InR* P2 promoter that were cloned into the pET101/D-TOPO vector

Forward primers	Sequence*	Reverse primers	Sequence	Amplicon size
D. melanogaster				
mInR_F -2816	**<u>CACC</u>**ATCCCATCCCAAATCCAAC	mInR_R-2525	TCGAGTGTAAAAAGTGCAGGGCAGA	339
mInR_F-1282	**<u>CACC</u>**TATTTTTTGCCTATGGT	mInR_R-955	CACAACTACTGCGGAGAAAT	363
mInR_F-982	**<u>CACC</u>**TGCTGTAACGCTCAAA	mInR_R-701	TTTATGCTTAAAACCCGATTC	317
mInR_F-782	**<u>CACC</u>**TTAGAAAACTGCAAAAT	mInR_R-395	GACAGACGCAGCTAAACTAG	424
mInR_F-503	**<u>CACC</u>**TATTTTCTTGAGTGGTTT	mInR_R-202	CAGTGCCAGTAGTTTCCATT	338
mInR_F-213	**<u>CACC</u>**GAGAGACCGAGACTGAG	mInR_R+49	CGACAGCGACAATAACAACACACT	302
D. simulans				
mInR_F-2816	**<u>CACC</u>**ATCCCATCCCAAATCCAAC	mInR_R-2525	TCGAGTGTAAAAAGTGCAGGGCAGA	337
sInR_F-1390	**<u>CACC</u>**TTCCGATTTTGTTGTTATTA	sInR_R-1028	TTTGCTTTTGGGCGTTACAG	402
sInR_F-1107	**<u>CACC</u>**TATGTCCCCAGATTGAA	sInR_-709	TTTATGCTTGAAACCCGATT	417
sInR_F-855	**<u>CACC</u>**AGAGTGCGGCTTTGTAGA	sInR_R-393	GCAGACAGACAGACGCAGCTAAACT	504
sInR_F-454	**<u>CACC</u>**ATTGTTAATTTGTTTTCATACC	s_InR_R-78	TTTCCTTCTTCTGCCACTTCTT	419
sInR_F-183	**<u>CACC</u>**TGGAAACTACTGGCACTG	sInR_+98	ATAGATACGCTCACCAACACACG	322
D. yakuba				
yInR_F-2825	**<u>CACC</u>**AGGGGGAGCAGACAACC	yInR_R-2452	TTTCAGACGATTAGCAGCGAGTTTTT	415
yInR_F-1393	**<u>CACC</u>**GCTGATTATTTTTGTGTT	yInR_R-1039	GGACGCTATTCCTTTTTGATGTT	377
yInR_F-1095	**<u>CACC</u>**TTCTACGCCTATATCCTC	yInR_R-798	CTCGCTTTCTAGGAACTCTATAATA	339
yInR_F-833	**<u>CACC</u>**CGCACAGTATTTGTATATT	yInR_R-521	AATTTGACCAATGACGAATACG	352
yInR_F-619	**<u>CACC</u>**TGTATGACGCAATCAATA	yInR_R-258	CTCAAAGCCCCAGTTCAGTT	398
yInR_F-255	**<u>CACC</u>**TTGTTTCTGCTCGGAACT	yInR_R+43	AGAATGGGGAGAACTACATAAGCAA	315
D. pseudoobscura				
pInR_F -2657	**<u>CACC</u>**ATTACAAGCGAATGAAACA	pInR_R -1418	GCGTGCCGAGTAAGAGTGAGAGGAG	392
pInR_F -1468	**<u>CACC</u>**GCCCTGGAACCTGGAAGA	pInR_R -1126	CTCTGCCGCTGCTGGTTCTGCTATT	384
pInR_F -1130	**<u>CACC</u>**CGTAATAACAAAAGCAG	pInR_R -819	CGTTCTGTCGTCCCTCTATTC	349
pInR_F -858	**<u>CACC</u>**GAGAACAAGAGCCACAAC	pInR_R -494	AGAGAAAAGGCGTGGTCAGAAA	403
pInR_F -514	**<u>CACC</u>**ATAAGCAAACCTTCAATAGA	pInR_R -196	GAGCAGACAGATAGACGCAAGT	359
pInR_F -207	**<u>CACC</u>**GCTCTTATTGTCCCTATC	pInR_R +286	TCTCTTCTTTCTCTGCGTCTTACAA	493
D. virilis				
vInR_F -3045	**<u>CACC</u>**TGCTTATTGTTGATTTTGTTAC	vInR_R +41	ACGCAAAAGTCAGCAGCAATAG	3130
vInR_F -1478	**<u>CACC</u>**ATATTATTGCTGTTGTGT	vInR_R -1167	GGAGGAGGCAGAAAACTTA	347
vInR_F -1243	**<u>CACC</u>**AACTTCAGTTAGGGTATA	vInR_R -803	CTGGATTACCTTTCACACTCA	478
vInR_F -867	**<u>CACC</u>**CGCACACATAAAAACATA	vInR_R -600	ACACAAAGAGCCGAAAGGAAC	306
vInR_F -615	**<u>CACC</u>**CAGAAAACTTGAGCATTA	vInR_R -240	AAATACATATCGAGGCAGACAAA	415
vInR_F -330	**<u>CACC</u>**CTCTGTTGTTTTCTTCCA	vInR_R +41	ACGCAAAAGTCAGCAGCAATAG	410

*The 4 nucleotides needed to clone the insert into the pET101/D-TOPO vector are indicated in bold and underlined
