# Supplementary material for: Characterization of dFOXO binding sites upstream of the Insulin Receptor P2 promoter across the Drosophila phylogeny
Source: PLoS One. 2017 Dec 4;12(12):e0188357. doi: 10.1371/journal.pone.0188357 (PMC5714339; doi:10.1371/journal.pone.0188357)
Supplement: S4 Table — (PDF) [file pone.0188357.s010.pdf]

S4 Table. Oligonucleotides used for RT-PCR experiments

gene	Forward Primer		Reverse Primer		Probe	
eGFP	eGFP_Fw496	AAGCAAAAGAACGGCATCAAA	eGFP_Rv554	CCGCCGTCTTCGATGTTG	eGFP_MGB519	CAACTTCAAGACCCGCC
InR_P2	InR_P2_Fw57	GTTATTGTCGCTGTCGTTGTTTTT	InR_P2_Rv116	CGCTCACCAACACATGCAA	InR_P2_MGB82	CCGCTGGCGCTTT
eIF-1A	eIF-1A_Fw265	TTCGCAAGAAGGTGTGGATTAA	eIF-1A_Rv319	TCACGCAAGCCCACCAA	eIF-1A_MGB288	CAGGGCGACATCAT
