# Supplementary material for: Characterization of dFOXO binding sites upstream of the Insulin Receptor P2 promoter across the Drosophila phylogeny
Source: PLoS One. 2017 Dec 4;12(12):e0188357. doi: 10.1371/journal.pone.0188357 (PMC5714339; doi:10.1371/journal.pone.0188357)
Supplement: S5 Table — (PDF) [file pone.0188357.s011.pdf]

**S5 Table.** Results of RT-PCR experiments

| | | | *eGFP* | | | | *InR* | | | | *eIF-1A* | | | |
|---|---|---|---|---|---|---|---|---|---|---|---|---|---|---|
| Experiment | Line | ng Total RNA | $C_t$ | $C_t$ Mean | $C_t$ StdDev | RNA ng of transcript | $C_t$ | $C_t$ Mean | $C_t$ StdDev | RNA ng of transcript | $C_t$ | $C_t$ Mean | $C_t$ StdDev | RNA ng of transcript |
| 1 | pGR | 200.00 | | | | | | | | | 16.227 | | | |
| | (no insert) | | 20.726 | | | | 25.444 | | | | 16.192 | | | |
| | | | 20.400 | 20.563 | 0.231 | | 25.413 | 25.428 | 0.022 | | 16.149 | 16.189 | 0.039 | |
| | | 50.00 | 22.227 | | | | 27.047 | | | | 18.166 | | | |
| | | | 22.475 | | | | 27.058 | | | | 18.201 | | | |
| | | | 22.297 | 22.333 | 0.128 | | 27.410 | 27.171 | 0.206 | | 18.183 | 18.183 | 0.017 | |
| | | 12.50 | 23.742 | | | | 29.207 | | | | 20.295 | | | |
| | | | 24.738 | | | | 28.994 | | | | 20.220 | | | |
| | | | 24.390 | 24.290 | 0.505 | | 29.275 | 29.159 | 0.146 | | 20.311 | 20.276 | 0.048 | |
| | | 3.13 | 26.621 | | | | 30.970 | | | | 22.366 | | | |
| | | | 26.013 | | | | 31.223 | | | | 22.190 | | | |
| | | | 25.906 | 26.180 | 0.386 | | 30.929 | 31.041 | 0.159 | | 22.264 | 22.273 | 0.088 | |
| | Dmut_ Pmut | 200.00 | 20.515 | | | | 25.038 | | | | 16.680 | | | |
| | | | 20.474 | | | | 25.104 | | | | 16.690 | | | |
| | | | 20.457 | 20.482 | 0.030 | 203.730 | 25.024 | 25.055 | 0.043 | 250.112 | 16.669 | 16.680 | 0.010 | 142.156 |
| | Dwt_Pmut | 200.00 | 20.637 | | | | 23.992 | | | | 16.391 | | | |
| | | | 20.476 | | | | 23.920 | | | | 16.257 | | | |
| | | | 20.294 | 20.469 | 0.172 | 205.692 | 23.886 | 23.932 | 0.054 | 570.188 | 16.301 | 16.316 | 0.068 | 182.116 |
| 2 | pGR | 200.00 | 20.724 | | | | 25.374 | | | | 16.297 | | | |
| | (no insert) | | 20.504 | | | | 25.342 | | | | 16.320 | | | |
| | | | 20.597 | 20.608 | 0.110 | | 25.417 | 25.379 | 0.053 | | 16.318 | 16.312 | 0.013 | |
| | | 50.00 | 22.280 | | | | 26.964 | | | | 18.387 | | | |
| | | | 22.405 | | | | 27.157 | | | | 18.398 | | | |
| | | | 22.327 | 22.337 | 0.063 | | 27.092 | 27.071 | 0.098 | | 18.415 | 18.400 | 0.014 | |
| | | 12.50 | 24.186 | | | | 29.143 | | | | 20.307 | | | |
| | | | 24.441 | | | | 29.169 | | | | 20.431 | | | |
| | | | 24.352 | 24.327 | 0.129 | | 29.070 | 29.127 | 0.051 | | 20.337 | 20.358 | 0.065 | |
| | | 3.13 | 26.879 | | | | 30.930 | | | | 22.435 | | | |
| | | | 26.583 | | | | 31.077 | | | | 22.281 | | | |
| | | | 26.216 | 26.559 | 0.332 | | 31.117 | 31.041 | 0.098 | | 22.400 | 22.372 | 0.081 | |
| | Dmut_Pwt | 200.00 | 19.290 | | | | 24.070 | | | | 15.835 | | | |
| | | | 19.049 | | | | 24.088 | | | | 15.837 | | | |
| | | | 19.353 | 19.231 | 0.160 | 478.943 | 24.089 | 24.083 | 0.011 | 483.825 | 15.867 | 15.846 | 0.018 | 280.881 |
| | mut_ChIP | 200.00 | 19.988 | | | | 24.622 | | | | 16.422 | | | |
| | | | 19.996 | | | | 24.798 | | | | 16.368 | | | |
| | | | 20.170 | 20.051 | 0.103 | 269.936 | 24.745 | 24.722 | 0.090 | 303.870 | 16.338 | 16.376 | 0.043 | 195.037 |
| 3 | pGR | 200.00 | 20.738 | | | | 25.321 | | | | 16.292 | | | |
| | (no insert) | | 20.470 | | | | 25.355 | | | | 16.259 | | | |
| | | | 20.535 | 20.581 | 0.140 | | 25.440 | 25.397 | 0.060 | | 16.280 | 16.277 | 0.017 | |
| | | 50.00 | 22.421 | | | | 26.964 | | | | 18.368 | | | |
| | | | 22.646 | | | | 27.056 | | | | 18.241 | | | |
| | | | 22.384 | 22.484 | 0.141 | | 27.314 | 27.111 | 0.181 | | 18.239 | 18.283 | 0.074 | |
| | | 12.50 | 24.400 | | | | 29.095 | | | | 20.440 | | | |
| | | | 24.320 | | | | 29.058 | | | | 20.303 | | | |
| | | | 24.386 | 24.369 | 0.043 | | 29.156 | 29.103 | 0.050 | | 20.353 | 20.366 | 0.069 | |
| | | 3.13 | 26.724 | | | | 31.123 | | | | 22.289 | | | |
| | | | 26.798 | | | | 30.439 | | | | 22.317 | | | |
| | | | 26.667 | 26.730 | 0.066 | | 30.949 | 30.837 | 0.356 | | 22.355 | 22.320 | 0.033 | |
| | wt | 200.00 | 17.840 | | | | 24.860 | | | | 15.911 | | | |
| | | | 17.748 | | | | 24.911 | | | | 15.866 | | | |
| | | | 18.145 | 17.911 | 0.208 | 1160.662 | 24.884 | 24.885 | 0.025 | 283.362 | 15.890 | 15.889 | 0.023 | 261.443 |

The expression of one reporter and two endogenous genes in five transgenic constructions was analyzed by RT-PCR. Samples were distributed between three experiments. Each experiment consisted on three replicates of each sample and all of them included samples from line pGR (with no insert) for four different total RNA dilutions to obtain the standard curve. Experiment 1 also included samples from two other transgenic lines (Dmut_Pmut and Dwt_Pmut). Samples from lines Dmut_Pwt and mut_ChIP, were tested in Experiment 2, and those from line wt in Experiment 3. Each experiment included blank samples that yielded in all cases Ct values ≥ 34.
